# Safety of Percutaneous Hepatic Perfusion with Melphalan in Patients with Unresectable Liver Metastases from Ocular Melanoma Using the Delcath Systems’ Second-Generation Hemofiltration System: A Prospective Non-randomized Phase II Trial

**DOI:** 10.1007/s00270-019-02177-x

**Published:** 2019-02-14

**Authors:** T. Susanna Meijer, Mark C. Burgmans, Marta Fiocco, Lioe-Fee de Geus-Oei, Ellen Kapiteijn, Eleonora M. de Leede, Christian H. Martini, Rutger W. van der Meer, Fred G. J. Tijl, Alexander L. Vahrmeijer

**Affiliations:** 1 Department of Radiology and Nuclear Medicine, Leiden University Medical Center, Postal Zone C2-S, Albinusdreef 2, 2300 RC Leiden, The Netherlands; 2 Department of Medical Statistics and Bioinformatics, Leiden University Medical Center, Leiden, The Netherlands; 3 Biomedical Photonic Imaging Group, University of Twente, Enschede, The Netherlands; 4 Department of Medical Oncology, Leiden University Medical Center, Leiden, The Netherlands; 5 Department of Surgery, Leiden University Medical Center, Leiden, The Netherlands; 6 Department of Anesthesiology, Leiden University Medical Center, Leiden, The Netherlands; 7 Department of Extra Corporal Circulation, Leiden University Medical Center, Leiden, The Netherlands

**Keywords:** Percutaneous hepatic perfusion, Chemosaturation, Melphalan, Liver metastasis, Melanoma

## Abstract

**Purpose:**

To investigate the safety and toxicity of percutaneous hepatic perfusion with melphalan (M-PHP) with the Delcath Systems’ second-generation (GEN 2) filter and compare the outcomes with historical data from studies using the first-generation filter.

**Materials and Methods:**

A prospective, single-arm, single-center phase II study was carried out including 35 patients with unresectable, histologically confirmed liver metastases from ocular melanoma between February 2014 and June 2017. Main exclusion criteria were extrahepatic disease and age > 75 years. M-PHP was performed with melphalan 3 mg/kg (maximum dose 220 mg). Safety and toxicity were assessed according to the Common Terminology Criteria for Adverse Events version 4.03.

**Results:**

A total of 67 M-PHPs were performed in 35 patients (median 2 procedures). Although hematologic grade 3/4 events were seen in the majority of patients (thrombocytopenia 54.5%, leukopenia 75.6%, neutropenia 66.7%, anemia (only grade 3) 18.1%), these were all well manageable or self-limiting. Of the non-hematologic grade 3 events (*n *= 14), febrile neutropenia (*n *= 3), pulmonary emboli (*n *= 2) and post-procedural hemorrhage (*n *= 2) were most common. A case of sepsis with bacterial pharyngitis was the only non-hematologic grade 4 event. Prior therapy for liver metastases was found to be a predictor of late grade 3/4 neutropenia with an odds ratio of 5.5 (95% CI 1.4–21.7).

**Conclusions:**

M-PHP using the GEN 2 filter has an acceptable safety and toxicity profile, and seems to reduce hematologic toxicity when compared to M-PHP with a first-generation filter. Prior therapy of liver metastases is a possible predictive factor in developing grade 3/4 hematologic toxicity.

## Introduction

The superiority of percutaneous hepatic perfusion with melphalan (M-PHP) over best available care in controlling liver disease in patients with metastases from ocular and cutaneous melanoma has been demonstrated in a phase III randomized controlled trial (RCT) [1]. In the Netherlands, M-PHP has recently been adopted as first-line treatment option for patients with metastatic ocular melanoma as they often present with unresectable metastases confined to the liver, and effective systemic therapies are lacking [2, 3]. M-PHP is also performed in patients with hepatic metastases from neuro-endocrine tumors, sarcomas and various types of carcinomas, as well as in patients with primary liver tumors [4–10].

Although M-PHP is well tolerated by most patients, adverse events (AEs) are not uncommon. Most notable are hematologic events due to bone marrow suppression. Bone marrow suppression results from the inability of hemofiltration cartridges to extract all melphalan, resulting in a limited amount of chemotherapeutics that reaches the systemic circulation. Reported percentages of hematologic events after M-PHP vary from 15 to 100% for anemia, 43–86% for neutropenia and 29–98% for thrombocytopenia [1, 4, 8–12].

In an attempt to reduce hematologic toxicity, various modifications were made to the original first-generation filter resulting in a second-generation (GEN 2) filter that became commercially available in 2012 [13]. A recent pharmacological study showed that the mean extraction rate of the GEN 2 hemofiltration system is 86%, which is approximately 10% higher than that of first-generation filters [13]. Although initial data indicate that using the GEN 2 filter may indeed reduce hematologic toxicity, this has never been evaluated prospectively [5, 10].

In 2014, a single-arm prospective phase II study was initiated to investigate M-PHP using the GEN 2 filter in patients with unresectable liver metastases from ocular melanoma. Although survival results are still pending, it is of clinical relevance to share the results on safety and toxicity in advance. The aim of this paper was to report all safety and toxicity results and compare these with historical data from studies on M-PHP using the first-generation filter [1, 6, 8, 11, 12]. Our data on the efficacy of M-PHP with the GEN 2 filter will be reported separately.

## Materials and Methods

### Study Design and Patients

This prospective, single-arm, single-center phase II study was approved by the Local Medical Ethics Committee of the Leiden University Medical Center and registered at www.trialregister.nl (trial identification NTR4112). Written informed consent was given by all patients.

Between February 2014 and June 2017, 35 patients with unresectable ocular melanoma metastases confined to the liver were enrolled. Histology specimens of liver metastases were obtained in all patients. Exclusion criteria are listed in Table 1. In case of enucleation, M-PHP was scheduled at least 4 weeks after surgery in order to prevent orbital bleeding complications as a result of per-procedural heparinization.

**Table 1:** Exclusion criteria

Laboratory test results	Other
APTT > 1.5 × ULN	Age < 18 or > 75 years
PT > 1.5 × ULN	Extrahepatic disease (on CECT or FDG-PET/CT)
Leukocytes < 3.0 × 10^9^/L	WHO performance status ≥ 2
Thrombocytes < 100 × 10^9^/L	Severe comorbidity precluding general anesthesia
Creatinine clearance < 40 ml/min	Diabetes with nephropathy
AST > 2.5 × ULN	Active infections
ALT > 2.5 × ULN	< 40% healthy liver tissue
Serum bilirubin > 1.5 × ULN	Other liver disease
ALP > 2.5 × ULN	Vascular anatomy impeding M-PHP
LDH > 2 × ULN	Intracranial lesions with propensity to bleed (on CT/MRI)
	Pregnancy

*ALP* alkaline phosphatase, *ALT* alanine aminotransferase, *APTT* activated partial thromboplastin time, *AST* aspartate aminotransferase, *CECT* contrast-enhanced computed tomography of chest and abdomen, *CT* computed tomography, *FDG*-*PET/CT* positron emission tomography with integrated non-contrast-enhanced computed tomography and 18F-2-fluoro-2-deoxy-d-glucose as radiotracer, *LDH* lactate dehydrogenase, *M*-*PHP* percutaneous hepatic perfusion with melphalan, *MRI* magnetic resonance imaging, *ULN* upper limit of normal, *PT* prothrombin time

### M-PHP Procedure

**Fig. 1:**
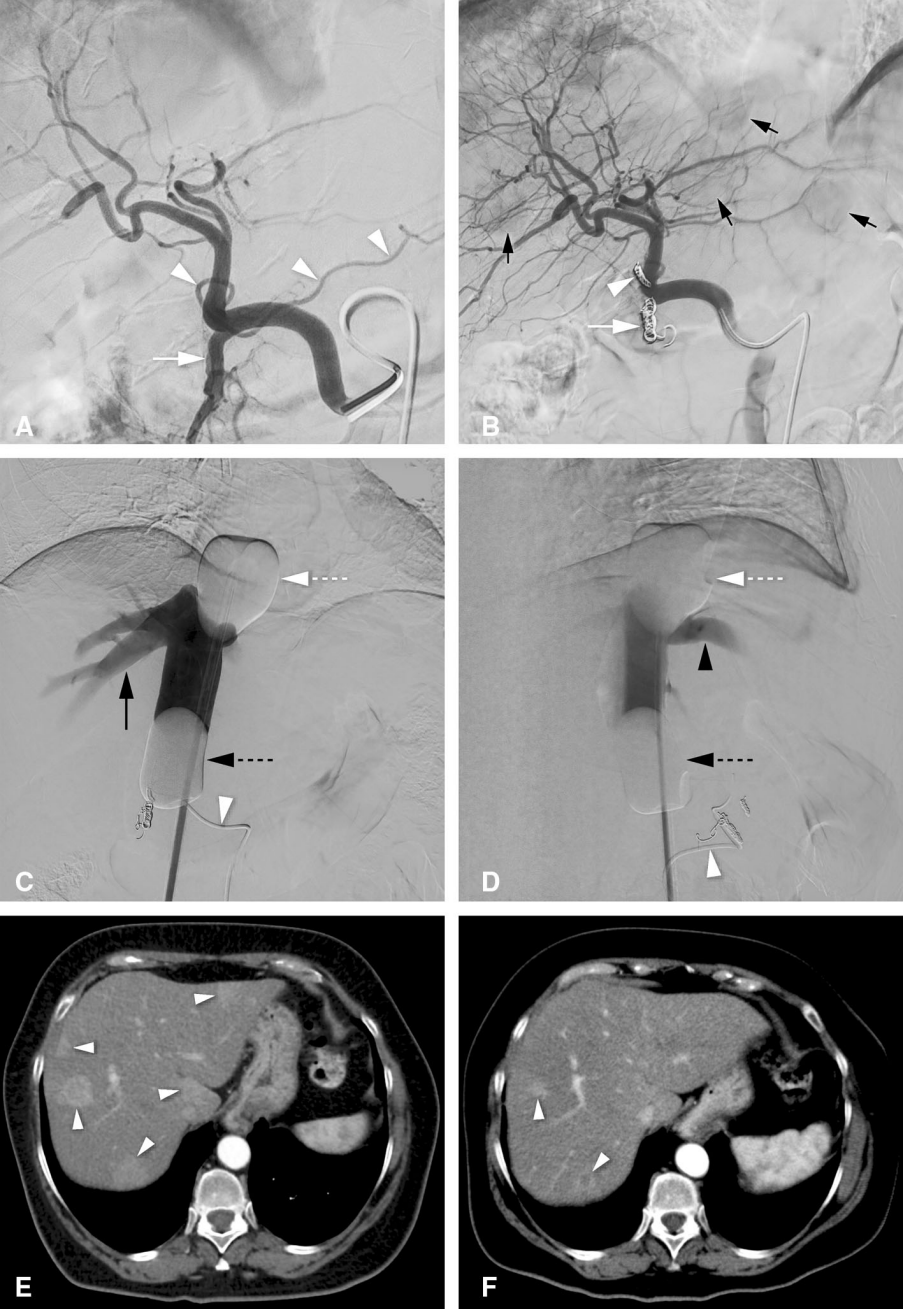
Hepatic vascular mapping and M-PHP in a 59-year-old female with bilobar hepatic metastases from uveal melanoma. **A** Angiographic image from the celiac trunk, showing a right gastric artery (white arrowheads) and gastroduodenal artery (white arrow) from the common hepatic artery. **B** Successful coiling of the right gastric artery (white arrowhead) and gastroduodenal artery (white arrow). Multiple hypervascular metastases are seen in both liver lobes (black arrows). **C**, **D** Posteroanterior and lateral images during venography, performed by manual injection of non-diluted contrast medium through side holes of the double-balloon catheter. The cranial balloon (dotted white arrow) was inflated at the atriocaval junction and the caudal balloon (dotted black arrow) in the infrahepatic portion of the inferior vena cava. Note the opacification of the right hepatic vein (black arrow) and middle hepatic vein (black arrowhead), while there was no leakage alongside the balloons. A microcatheter (white arrowhead) was placed into the hepatic artery proper for the infusion of melphalan. **E** Axial CT image in arterial phase before treatment showing five hepatic metastases (white arrowheads). **F** Axial CT image in arterial phase after two cycles of M-PHP showing reduction in size of two metastases in the right lobe. The other three metastases showed a complete radiological response

All patients underwent angiographic evaluation of the hepatic arteries approximately one week prior to M-PHP. If deemed necessary, hepatico-enteric anastomoses (e.g., gastroduodenal and right gastric artery) were embolized to prevent inadvertent leakage of melphalan (Fig. 1).

All M-PHP procedures were performed in an angiographic suite under general anesthesia by an interventional radiologist, anesthesiologist and extracorporeal perfusionist. A cannula in the radial artery and triple lumen line in the left internal jugular vein (IJV) were placed to enable continuous monitoring of the arterial and central venous pressure, and infusion of sympathomimetics and fluids. Access to the right IJV (10-F sheath), right common femoral vein (CFV, 18-F sheath) and left common femoral artery (5-F sheath) was created. Heparin was administered at an initial dose of 300 U/kg, and an activated clotting time of ≥ 450 s was maintained throughout the entire procedure. After hepatic angiograms were obtained, the tip of a 2.4F or 2.7F microcatheter was placed into the hepatic artery at the intended location of infusion. A 16-F double-balloon catheter (Isofuse Isolation Aspiration Catheter, Delcath Systems Inc, New York, NY, USA) was placed in the inferior vena cava (IVC) via the right CFV. The cranial and caudal balloons were inflated to occlude the atriocaval junction and infrahepatic portion of the IVC, respectively, to prohibit leakage of melphalan into the systemic circulation. A venogram was obtained through the injection port of the double-balloon catheter to confirm correct positioning (Fig. 1). Then, the entire dose of melphalan was infused into the proper hepatic artery or split and infused in the right and left hepatic artery in a selective lobar approach. Melphalan-enriched blood was aspirated through catheter fenestrations in a segment between the two balloons, pumped through an extracorporeal hemofiltration system including two activated carbon filters and returned to the patient through the sheath in the right IJV. After the infusion was completed, extracorporeal filtration was continued for 30 min (washout period) to allow clearance of melphalan from the liver. At the end of the procedure, the coagulation status was corrected with protamine sulfate 3 mg/kg, the arterial sheath was removed and hemostasis was achieved using a closure device. For a more extensive description, see the paper by Burgmans et al. [4].

All patients underwent two cycles of M-PHP at a 6–8-week interval (9 weeks in one patient), except for patients with progressive disease after the first treatment, unacceptable AEs or patients’ reluctance to undergo further treatment. All first, M-PHPs were performed with 3 mg melphalan/kg and a maximum dose of 220 mg. In case of grade 3/4 hematologic toxicity, the melphalan dose for the second M-PHP was reduced with 20–25%.

### Post-procedural Management

Patients were admitted to the hospital for 2–3 days, with the first night spent at the post-anesthesia care unit. Venous sheaths were removed 4–6 h after completion of the treatment, and hemostasis was achieved using manual compression. Patients with platelet levels of < 50 × 10^9^/L received a platelet transfusion prior to removal of the sheaths. Full blood count and liver function tests were performed daily, and leukocyte differential count was performed on day 2. Within 72 h of the M-PHP, patients received a single injection of PEGylated granulocyte colony-stimulating factor (G-CSF, pegfilgrastim 6 mg). Preventive administration of pegfilgrastim was not incorporated in the original study protocol, but introduced after the first M-PHP in patient number 2 was complicated by severe neutropenia and sepsis due to bacterial pharyngitis.

### Post-procedural Follow-Up

Blood tests (full blood count, leukocyte differential count, liver function) were performed on days 7, 9, 11, 14 and 16. Blood tests and contrast-enhanced computed tomography (CECT) of chest and abdomen (including arterial phase of the liver) were performed 4–8 weeks after the first and second M-PHPs, and then every 3 months in the first year and every 6 months thereafter until disease progression occurred. In one patient, first imaging after the second M-PHP was performed 10 weeks post-treatment. When lesions were difficult to visualize on CECT, additional magnetic resonance imaging (MRI) of the liver was performed.

### Endpoints and Definitions

Primary safety endpoint was the number of serious adverse events (SAEs) occurring within 30 days after M-PHP. A SAE was defined as a serious complication resulting in death, a life-threatening situation, prolonged hospital admission or readmission. SAEs were reported according to the Common Terminology Criteria for Adverse Events version 4.03 (CTCAE v4.03) [14]. Secondary safety endpoints included all other AEs and also reported according to CTCAE v4.03 and technical success.

Hematologic and hepatic events were reported as early (days 0–3) and late events (days 4–30) as they were thought to have a different underlying cause; early events were considered to be related to the procedure itself (i.e., hemolysis by the filtration system and/or hemodilution), whereas late events were attributed to systemic exposure to melphalan.

Technical success was defined as successful administration of all prescribed melphalan with completion of the washout period of 30 min.

### Statistical Analysis

Statistical analyses were performed using SPSS 23.0 (SPSS Inc., Chicago, IL, USA). Post-treatment laboratory test results were compared to pretreatment results using the Wilcoxon signed-rank test. To avoid type 1 error due to multiple testing, Bonferroni corrections were performed. A *p* value less than 0.05 was considered statistically significant.

To investigate the effect of possible risk factors on late hematologic toxicity, a multivariate generalized linear mixed model with predictor treatment with previous therapy, patient characteristics and procedure-related variables as random effects was estimated. Analyzed patient characteristics included age, gender and BMI. Procedure-related variables included total melphalan dose, melphalan dose/kg body weight, melphalan dose/ml liver volume, type of double-balloon catheter (50 or 62 mm) and total filtration time.

## Results

### Patients and M-PHP Procedures

A total of 67 M-PHP procedures were performed in 35 patients. Baseline characteristics are reported in Table 2. Most patients (77.1%) received two M-PHPs as per protocol. In 17.1% of patients, only one M-PHP was performed. One patient had three and one patient had four M-PHPs; they received additional treatments after hepatic progression occurred following a progression-free interval of > 6 months. All patients were included in the analysis.

**Table 2:** Baseline patient characteristics (*n* = 35)

Parameters	
Gender [*n* (%)]	
Men	16 (45.7)
Women	19 (54.3)
Age at inclusion [years; median (range)]	59 (42–71)
BMI [kg/m^2^; median (range)]	24.8 (20.4–32.2)
Interval between diagnosis of primary tumor and liver metastases [months; median (range)]	28 (0–71)
Type of metastases [*n* (%)]	
Synchronous	4 (11.4)
Metachronous	31 (88.6)
Mutations in liver metastases	
GNAQ	21 (60.0)
GNA11	12 (34.3)
No GNAQ/GNA11	2 (5.7)
Prior therapy for liver metastases [n (%)]	
Systemic therapy^a^	8 (22.9)
Regional therapy^b^	4 (11.4)
Regional and systemic therapy	2 (5.7)
No prior therapy	21 (60.0)
Number of metastases^c^ [*n* (%)]	
1–5	9 (25.7)
6–9	8 (22.9)
≥ 10	18 (51.4)
Liver function tests	
LDH level (U/L)	196 (78–657)
AST level (U/L)	24 (12–89)
ALT level (U/L)	26 (8–82)
Bilirubin level (µmol/L)	6 (3–20)

*ALT* alanine transaminase, *AST* aspartate transaminase, *BMI* body mass index, *GNAQ* guanine nucleotide-binding protein G(q) subunit alpha, *GNA11* guanine nucleotide-binding protein G(Y) subunit alpha-11, *LDH* lactate dehydrogenase

^a^Randomized phase II SUMIT trial (selumetinib with dacarbazine vs. placebo), ipilimumab, phase I AEB071 study (protein kinase C inhibitor), dendritic cell therapy

^b^Radiofrequency ablation and/or metastasectomy

^c^Based on diagnostic imaging

In 92.5% (62/67) of cases, M-PHP was technical successful. Five procedures were discontinued early because of filter clotting (*n* = 3), insufficient sealing of the cranial balloon at its atriocaval junction (*n* = 1) and transient cardiac ischemia (*n* = 1). In one patient with heparin-induced thrombocytopenia, filter clotting occurred twice despite using argatroban as alternative anticoagulant during the second procedure.

Median melphalan dose in all technically successful M-PHPs during the first and second cycle was 220 mg (range 170–220) and 178 mg (range 140–220), respectively. Median dose per kilogram body weight was 2.7 mg/kg (range 2.2–3.1) for the first and 2.4 mg/kg (range 1.8–3.4) for the second cycle.

### Safety

A total of 14 SAEs were recorded (Table 3). No deaths occurred. One patient developed per-procedural cardiac ischemia which was managed conservatively and resolved without sequelae. There were five cases of prolonged hospital stay (4–5 days) and eight readmissions (median hospital stay of 6 days, range 1–15).

**Table 3:** Serious adverse events in all M-PHPs (*n* = 67)

Serious adverse events	*n* = 14
Death	0
Potential life-threatening situation during M-PHP	1
Transient cardiac ischemia (*n* = 1)^a,b^	
Prolonged hospital admission	5
Post-procedural hypotension (asymptomatic) (*n* = 1)	
Peri-procedural difficulties with oxygenation (*n* = 1)^c,d^	
Post-procedural ECG changes (asymptomatic) (*n* = 1)^e^	
Pulmonary emboli (*n* = 1)^f^	
Nausea/vomiting with mild hypokalemia (*n* = 1)	
Readmission	8
Sepsis with bacterial pharyngitis and retropharyngeal abscess (*n* = 1)^c,g^	
Pulmonary emboli (*n* = 1)^c,f^	
Vaginal hemorrhage with grade 2 anemia (*n* = 1)	
Febrile neutropenia (*n* = 2)^h^	
Febrile neutropenia with mucositis/esophagitis (*n* = 1)	
Prostatitis (*n* = 1)^b^	
Abdominal pain (unknown cause) (*n* = 1)	

*ECG* electrocardiographic, *M*-*PHP* percutaneous hepatic perfusion with melphalan

^a^No second M-PHP, physicians’ decision

^b^Managed conservatively, patient resolved without sequelae

^c^No second M-PHP, patients’ decision

^d^Managed with prolonged intubation/ventilation and administration of norepinephrine, troponins not elevated

^e^Probably as a result of grade 2 anemia, troponins not elevated

^f^Symptomatic patient, successfully treated with low molecular weight heparin

^g^Treated with intravenous antibiotics and immunoglobulins, followed by aspiration of the retropharyngeal abscess

^h^In same patient

Grade 3/4 hematologic events were seen in the majority of patients with leukopenia (75.6%) and lymphocytopenia (84.8%) being most common (Table 4). Although grade 3/4 leukopenia and neutropenia were only observed after day 3, grade 3/4 thrombocytopenia and lymphocytopenia and grade 3 anemia were seen in both the early and late phases. Grade 4 anemia did not occur. Hematologic events in all technically successful M-PHPs are further specified in Table 5.

**Table 4:** Overview of grade 3/4 hematologic events in all patients that received at least one technically successful M-PHP (*n* = 33)

	Overall (0–30 days) [*n* (%)]	Early events (0–3 days) [*n* (%)]	Late events (4–30 days) [*n* (%)]
Grade 3/4 anemia^a^	6 (18.1)	1 (3.0)	5 (15.2)
Grade 3/4 thrombocytopenia	18 (54.5)	4 (12.1)	17 (51.5)
Grade 3/4 leukopenia	25 (75.6)	0 (0.0)	25 (75.6)
Grade 3/4 neutropenia	22 (66.7)	0 (0.0)	22 (66.7)
Grade 3/4 lymphocytopenia	28 (84.8)	20 (66.7)^b^	23 (69.7)

^a^Only patients with grade 3 anemia

^b^In 20/30 patients; in 3 patients, lymphocyte count was not available in the early phase

**Table 5:** Hematologic and hepatic events according to CTCAE v4.03 in all technically successful M-PHPs (*n* = 62)

		All grades *n* (%)	Grade 3 *n* (%)	Grade 4 *n* (%)
Hematologic events				
Anemia	*Early*	62 (100.0)	1 (1.6)	–
	*Late*	61 (98.4)	5 (8.1)	–
Thrombocytopenia	*Early*	58 (93.5)	6 (9.6)	–
	*Late*	45 (72.6)	11 (17.7)	11 (17.7)
Leukopenia	*Early*	10 (16.1)	–	–
	*Late*	45 (72.6)	9 (14.5)	22 (35.5)
Neutropenia	*Early*	–^a^	–	–
	*Late*	34 (55.7)^b^	2 (3.3)	26 (42.6)
Lymphocytopenia	*Early*	42 (93.3)^c^	17 (37.8)	5 (11.1)
	*Late*	48 (81.4)^d^	23 (39.0)	13 (22.0)
Hepatic events				
ALT increased	*Early*	34 (54.8)	–	–
	*Late*	36 (58.1)	–	–
AST increased	*Early*	43 (69.4)	–	–
	*Late*	21 (35.0)^e^	–	–
Bilirubin increased	*Early*	5 (8.8)^f^	–	–
	*Late*	6 (10.0)^g^	–	–

^a^In 44 M-PHPs, ^b^ In 61 M-PHPs, ^c^ In 45 M-PHPs, ^d^ In 59 M-PHPs, ^e^ In 60 M-PHPs, ^f^ In 57 M-PHPs, ^g^ In 60 M-PHPs

**Fig. 2:**
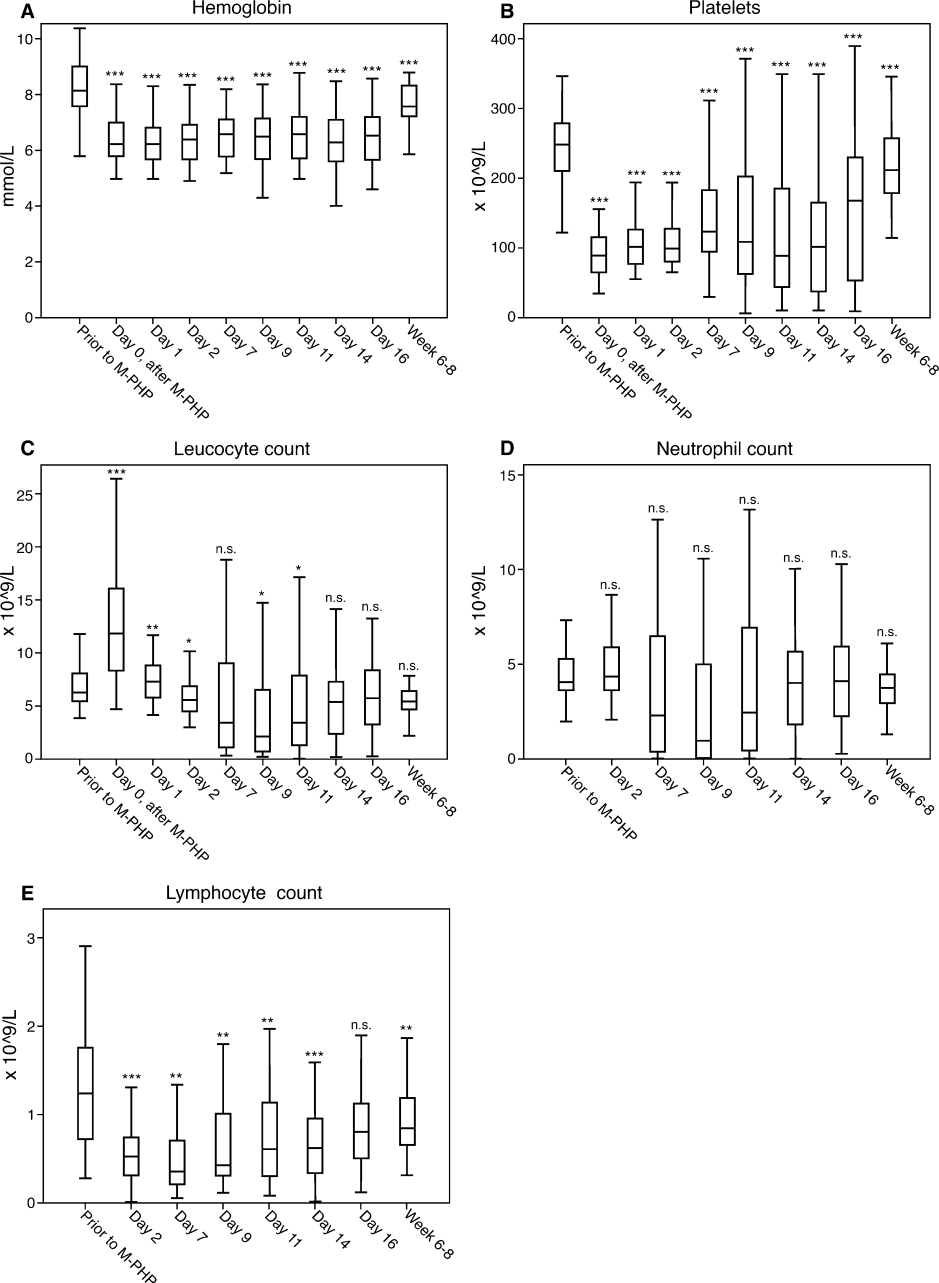
Laboratory values to evaluate hematologic function, from prior to M-PHP until 6–8 weeks after treatment (**A**–**E**). Post-procedural laboratory results were compared with pre-procedural results using the Wilcoxon signed-rank test. ***p < 0.001, **p < 0.01, *p < 0.05, *n.s.* not significant

**Fig. 3:**
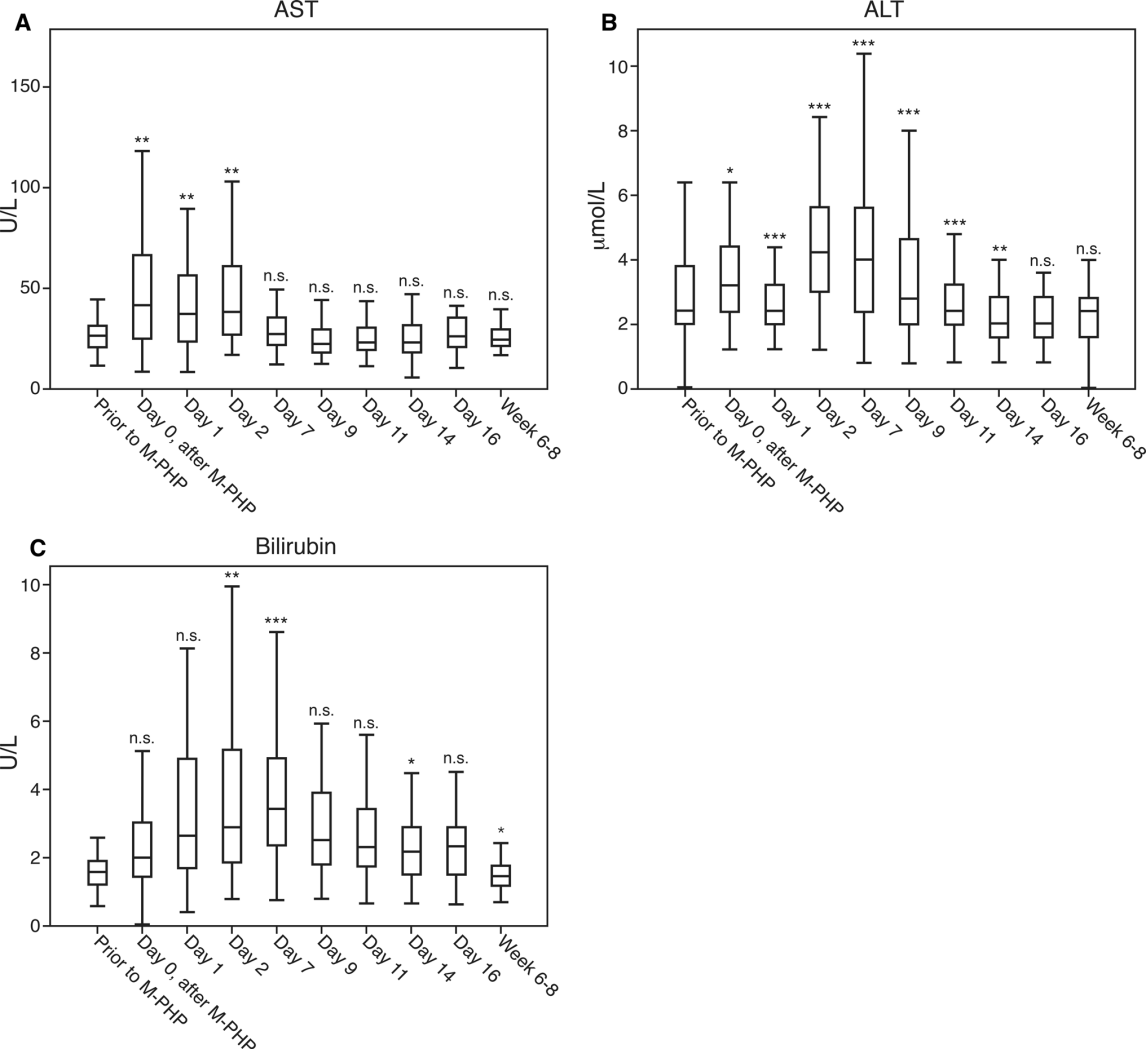
Laboratory values to evaluate hepatic function, from prior to M-PHP until 6–8 weeks after treatment (**A**–**C**). Post-procedural laboratory results were compared with pre-procedural results using the Wilcoxon signed-rank test. ***p < 0.001, **p < 0.01, *p < 0.05, *n.s.* not significant

There was a significant decrease in hemoglobin and platelet levels, and lymphocyte count during the early phase (*p* < 0.001, Fig. 2). The nadir for leukocytes, neutrophils and lymphocytes was reached at days 9, 9 and 7, respectively. Although there were no grade 3/4 hepatic events (Table 5), the increase of aminotransferases indicated some degree of hepatic toxicity (*p* < 0.01, Fig. 3).

All non-hematologic and non-hepatic complications are listed in Table 6. There were 14 grade 3 events of which post-procedural hemorrhage requiring transfusion (*n* = 2), pulmonary emboli (*n* = 2) and febrile neutropenia (*n* = 3) were most common. A case of sepsis with bacterial pharyngitis and retropharyngeal abscess was the only non-hematologic grade 4 event.

**Table 6:** Non-hematologic and non-hepatic complications in all M-PHPs (*n* = 67), reported according to CTCAE v4.03. The serious adverse events (see Table 3) are also incorporated

Complications	All grades (*n*)	Grade 3 (*n*)	Grade 4 (*n*)
Post-procedural hemorrhage	11^a^	2^b^	–
Generalized edema and/or pleural effusion^c^	8	–	–
Fever^d^	7	–	–
Nausea	7	1	–
Abdominal pain	4	1	–
Alopecia	3	–	–
Diarrhea	2	–	–
Pulmonary emboli	2	2	–
Febrile neutropenia	3	3	–
Sepsis with bacterial pharyngitis and retropharyngeal abscess	1	–	1
Cardiac ischemia during M-PHP	1	1	–
Post-procedural hypotension	1	1	–
Post-procedural ECG changes	1	1	–
Bladder infection	1	–	–
Cystitis, non-infective	1	–	–
Prostatitis	1	1	–
Peri-procedural difficulties with oxygenation	1	1	–
Upper respiratory infection	1	–	–
Vulval infection	1	–	–
Hyperglycemia	1	–	–
Total	58	14	1

^a^Bleeding from puncture site groin (*n *= 6), false aneurysm from puncture site groin (*n* = 2), hyposphagma in unaffected eye (*n* = 1), epistaxis (*n* = 1), vaginal hemorrhage (*n* = 1)

^b^Epistaxis and vaginal hemorrhage requiring platelet transfusion and readmission with transfusion of platelets and red blood cells, respectively

^c^Due to overhydration, only reported if treatment with diuretics was required

^d^Temperature 38–39 °C during admission, no signs of infection

### Transfusions and Antibiotics

In the early phase, three patients received one unit of platelets prior to the removal of vascular sheaths. RBC transfusions were not required. In the late phase, 8/33 patients (24.2%) received a mean of 1.6 platelet units and 5/33 patients (15.1%) received a mean of 2.4 RBC units. Platelets were transfused because of symptomatic thrombocytopenia (one patient with epistaxis) or platelet count < 10 × 10^9^/L (seven patients). In total, 9/33 patients (27.2%) received blood transfusions, with some patients receiving both RBC and platelet transfusions.

Antibiotics were given in seven patients on eight occasions for the following: sepsis with bacterial pharyngitis and retropharyngeal abscess (*n* = 1), mucositis/esophagitis and febrile neutropenia (*n* = 1), febrile neutropenia (*n* = 2), prostatitis (*n* = 1), bladder infection (*n* = 1), vulval infection (*n* = 1) and upper respiratory infection (*n* = 1).

### Predictive Factors for Late Hematologic Toxicity

The only variable that was found to be a predictor of late grade 3/4 neutropenia was prior therapy for liver metastases (systemic and/or local therapy) with an odds ratio of 5.5 (95% CI 1.4–21.7).

## Discussion

The results of this study show that grade 3/4 hematologic events are common after M-PHP, even with the GEN 2 filter. All events, however, were well manageable or self-limiting. Hematologic and hepatic toxicity percentages are significantly lower compared to studies using first-generation filters (see below). Prior therapy of liver metastases might be a predictor in developing late grade 3/4 neutropenia after M-PHP in ocular melanoma patients.

In a RCT by Hughes et al., 65 patients with ocular or cutaneous melanoma were treated with at least one M-PHP (median of three procedures) using the first-generation filter [1]. Similar to the current study, hematologic complications were categorized as early (days 0–3) or late (days 4–30) events enabling a direct comparison of toxicity in M-PHP with the GEN 2 and first-generation filter. We reported lower percentages of early grade 3/4 anemia (3.0% vs. 60.0%) and thrombocytopenia (12.1% vs. 74.3%). This indicates that the GEN 2 filter causes less damage to blood cells than first-generation filters. In addition, the lower rates of late grade 3/4 anemia (15.2% vs. 91.4%), thrombocytopenia (51.5% vs. 80.0%) and neutropenia (66.7% vs. 85.7%) in the current study strongly suggest that there is less bone marrow suppression due to a higher mean filter efficiency in the GEN 2 filter. Our patients even received a higher total dose of melphalan as a dose of 3 mg/kg actual body weight was used compared to 3 mg/kg ideal body weight in the RCT (in our population, median actual and ideal body weight was 77 kg and 66 kg, respectively). In addition, the current study protocol differed from Hughes’ protocol in that G-CSF was used as preventive drug in virtually all patients, whereas Hughes et al. only administered G-CSF when indicated. This may have contributed to the differences in observed neutropenia.

In a recent retrospective study by Kirstein et al., 29 patients received a median of two M-PHPs using the GEN 2 filter [10]. Although they reported higher percentages of grade 3/4 thrombocytopenia (89.7% vs. 54.5%) and anemia (41.3% vs. 18.1%), grade 3/4 neutropenia was reported less frequently (34.5% vs. 66.7%). Interestingly, this occurred despite the relatively limited use of G-CSF in only 38% of patients. We hypothesize that severe neutropenia was observed more often in the current study due to frequent blood testing that was performed after hospital discharge in all patients including asymptomatic patients.

To our knowledge, lymphocyte cell counts following M-PHP have not been reported in the literature before. As the decrease in cell count for other blood cells was less substantial, the early lymphocytopenia may have (partially) resulted from a cause other than direct damage by the filter that still needs clarification.

In this study, increase of aminotransferases was observed in the majority of patients but was mild and resolved within 16 days after the procedure in all cases. Reported percentages of grade 3/4 transaminitis vary from 6 to 20% in ocular melanoma patients to 41% in a diverse study population including patients with primary liver tumors which often have an underlying liver disease, such as fibrosis or cirrhosis [1, 10, 11].

Minor bleeding events were quite common and appeared in about 30% of patients. The only two events (epistaxis and vaginal hemorrhage) that required therapy, and were therefore classified as grade 3 events, both occurred more than 1 week after M-PHP.

Thromboembolic events have been reported before [1, 11, 12]. In our study, two patients were diagnosed with symptomatic pulmonary emboli on the first and 17^th^ day after M-PHP. Both patients were successfully treated with low molecular weight heparin (LMWH). LMWH was not routinely prescribed, as patients were usually ambulant on day 2 after M-PHP.

Prior therapy for liver metastases was found to be associated with late grade 3/4 neutropenia, but with a wide confidence interval. Other risk factors were not significantly associated with the outcome. Larger studies are needed to study the effects of prognostic factors on toxicity.

This study is limited by its small sample size, which can be explained by the rarity of the disease. Secondly, as there was no control arm, we were only able to compare our results with historical cohorts. Thirdly, systemic toxicity may not only be attributed to incomplete filtration by the filters. Other factors may play a role, such as insufficient sealing of the balloons or chemotherapeutics reaching the systemic circulation through venous collaterals. Finally, comparison of our results with other studies was complicated by protocol differences concerning the administration of G-CSF.

## Conclusions

This study suggests that hematologic toxicity after M-PHP can be reduced by using the GEN 2 filter instead of a first-generation filter. Although grade 3/4 hematologic events were still observed in the majority of patients, they were all well manageable or self-limiting. Prior therapy of liver metastases may be a risk factor for grade 3/4 hematologic toxicity after M-PHP in ocular melanoma patients.

## Abbreviations

AE: Adverse event
CECT: Contrast-enhanced computed tomography
CFV: Common femoral vein
CTCAE v4.03: Common terminology criteria for adverse events version 4.03
G-CSF: Granulocyte colony-stimulating factor
GEN 2: Second-generation
IVC: Inferior vena cava
IJV: Internal jugular vein
M-PHP: Percutaneous hepatic perfusion with melphalan
MRI: Magnetic resonance imaging
RBC: Red blood cell
RCT: Randomized controlled trial
SAE: Serious adverse event
